# Use of ^18^F-sodium fluoride bone PET for disability evaluation in ankle trauma: a pilot study

**DOI:** 10.1186/s12880-018-0277-1

**Published:** 2018-09-20

**Authors:** Tae Joo Jeon, Sungjun Kim, Jinyoung Park, Jung Hyun Park, Eugene Y. Roh

**Affiliations:** 1 Department of Nuclear Medicine, Gangnam Severance Hospital, Yonsei University College of Medicine, Seoul, South Korea; 2 Department of Radiology, Gangnam Severance Hospital, Yonsei University College of Medicine, Seoul, South Korea; 3 Department of Rehabilitation Medicine, Gangnam Severance Hospital, Rehabilitation Institute of Neuromuscular Disease, Yonsei University College of Medicine, Seoul, South Korea; 4 Division of PM&R, Department of Orthopaedic Surgery, Stanford University School of Medicine, Stanford, CA 94063 USA; 5 Department of Rehabilitation Medicine, Gangnam Severance Hospital, Yonsei University College of Medicine, 211 Eonjuro, Gangnam-gu, Seoul, 06273 South Korea

**Keywords:** Ankle injuries, Disability evaluation, PET-CT, Positron emission tomography, Range of motion

## Abstract

**Background:**

There are no objective and accurate rating tools for permanent impairment of traumatized ankles. The purpose of this study is to assess the role of 18F-Sodium fluoride (18F-NaF) positron emission tomography-computed tomography (PET/CT) bone scans in evaluating patients with limited ankle range of motion (ROM) after trauma.

**Methods:**

18F-NaF PET/CT was performed in 121 patients (75 men, 46 women; mean age: 45.8) who had ROM < 70% of normal after trauma affecting ankles. Metabolic target volume (MTV), the sum of voxels with standardized uptake value (SUV) > 2.5 was automatically obtained from the 3D volume that included the ankle joint. The maximum & mean SUV (SUVmax & SUVmean), and the total lesion activity (TLA) were measured.

**Results:**

The median period from injury to performing 18F-NaF PET/CT was 290 days. The causes of injury were as follows: fracture (*N* = 95), Achilles tendon rupture (*N* = 12), and ligament injury (*N* = 12). Hot uptake in the ankle was seen in 113 of 121 patients. The fracture group had higher SUVmax, SUVmean, and TLA values than the non-fracture group. More limited ROM correlated with higher hot-uptake parameters (SUVmax, SUVmean, TLA). In subgroup analysis, the same correlations were present in the fracture, but not in the non-fracture group.

**Conclusions:**

18F-NaF PET/CT can provide considerable information in impairment evaluations of limited ankle ROM, particularly in fracture around the ankle. Thus, 18F-NaF bone PET/CT may provide an additional option as an objective imaging tool in disability assessment after ankle injury.

## Background

Limitation of range of motion (ROM) in the ankle is common after ankle trauma, even after the time has passed for maximum medical improvement (MMI). Ankle stiffness causes difficulties in walking and restrictions in the activities of daily living, which is worse when it is accompanied by pain. The objective and accurate rating of permanent impairment is very important in workers’ compensation programs or automobile insurance, to determine an appropriate level of financial compensation [1, 2]. However, the impairment of ROM in the ankle joint is difficult to evaluate because it is based on direct observations, such as the patients’ own activities, which can be subjective and are easily influenced by pain or by their motivations to improve, rather than on objective measurement [3]. Radiologic studies performed immediately after injury are helpful to provide information about the severity of the trauma; however, after achieving MMI, it is impossible to find a correlation between the functional impairment of the ankle joint (pain or limitation of ROM) and the latest objective radiologic assessments (simple plain radiography, computerized tomography [CT], or magnetic resonance imaging).

Impairment of the ankle joint, such as contracture or limitation of ROM, can occur after traumatic injury; its pathomechanism may be explained by the concept of posttraumatic osteoarthritis (PTA) [4]. Ankle PTA after intra-articular injury could be related to the initial articular cartilage and subchondral bone damage, inadequate reduction of joint surfaces, or complications in the healing process [4–6]. Extra-articular injuries not involving joint structures could also cause end-stage ankle PTA by chronic instability or the long-term effects of posttraumatic malalignment [4, 7]. Furthermore, fibrosis of the soft tissue can play a role in post-traumatic joint contractures [8]. Posttraumatic Immobilization induces limitation of ROM by disuse osteoporosis and reversible bone loss with increased osteoclastic bone resorption in the mechanically unloaded bone [9]. However, to date, no biomarker has been available to represent ROM of the joint after injury.

Bone scanning using ^18^F-sodium fluoride (18F-NaF) was performed by Blau et al. in 1962, [10] but this was rapidly replaced by technetium-99 m (^99m^Tc)-labeled bone imaging agents after the introduction of gamma cameras equipped with a thallium-doped sodium iodide (NaI [Tl]) crystal. However, as positron emission tomography (PET) cameras came into use, interest in 18F-NaF bone scanning has renewed [11]. The uptake mechanism of 18F-NaF and ^99m^Tc-labeled diphosphonate is essentially the same, involving chemisorption, and the amount of bone accumulation depends on blood flow and the exposed bone surface [12]. However, negligible plasma protein binding, and the rapid blood and renal clearance of NaF permits earlier image acquisition after tracer administration. Therefore, 18F-NaF bone scanning has several advantages, such as enabling high spatial resolution, attenuating correction, allowing 3D tomographic imaging, as well as hybrid PET/CT imaging [11, 13]. Similar to ^99m^Tc agents, 18F-NaF has been mainly used for evaluation of bone metastasis; however, these high resolution tomographic images, with their corresponding CT images, are also very useful for evaluation of benign joint disease, such as enthesopathy, degenerative joint disease, and osteophytosis [14].

Impairment of ankle ROM after trauma has commonly been evaluated by physical examination by a doctor or therapist, which is subjective, rather than objective quantitative evaluation of images. In this study, the role of 18F-NaF bone PET/CT in the evaluation of impairment in trauma patients with limited ankle ROM was assessed.

## Methods

### Patients

Between September 2013 and March 2017, 121 patients (75 men and 46 women, mean age: 45.8 years; range: 17–75 years) were recruited into the study (Table 1); these patients had limited ankle ROM at least 6 months after traumatic injury affecting their ankles. The etiology of injury varied: 1) fracture with intra-articular involvement of the tibiotalar joint, 2) fracture without intra-articular involvement, 3) ligament injury, 4) Achilles tendon rupture, and 5) others. These individuals attended the outpatients’ clinic of a university hospital for an evaluation of the impairment of their ankles. For the impairment rating, the ROM of the ankle joint was measured according to “*AMA Guides to the Evaluation of Permanent Impairment*” [15]. Images of the ankles were obtained by plain radiography and 18F-NaF PET/CT. The study was approved by the institutional review board of our institute, and written informed consent was obtained from all study participants.

**Table 1:** Demographic data of 121 patients

Sex	
Male	75
Female	46
Mean Age (range)	45.8 (17–75)
Etiology of Injury	
Fracture with intra-articular involvement of the tibiotalar joint	54
Fracture without intra-articular involvement	41
Ligament injury	12
Achilles tendon rupture	12
Others	3

### 18F-NaF bone PET/CT imaging

18F-NaF bone PET/CT imaging was conducted in accordance with *SNM practice guidelines for Sodium F-18 Fluoride PET/CT bone scan 1.0* [16]. Patient fasting was not required, and 5.18 MBq/kg (0.14 mCi/kg) of 18F-NaF was injected. Regional PET with non-contrast-enhanced CT for attenuation correction was performed consecutively, 60 min after the injection of 18F-NaF, by means of a dedicated PET/CT system (Biograph mCT; Siemens Healthcare, Munich, Germany). The parameters for CT were 120 kVp, effective mAs controlled by Care Dose 4D software, 0.5-s gantry rotation, and 0.6-mm collimation. The kernel for CT reconstruction was the B60f sharp-type. 18F-NaF PET images so acquired were reconstructed using True X and time of flight (TOF), ultra-high definition (HD)-PET.

### Imaging analysis

18F-NaF PET/CT images of 121 patients with limited ROM of the ankle were reviewed using the dedicated software for PET/CT workstation (Syngo VE32B, Siemens AG). Metabolic target volume (MTV), the sum of voxels with a standardized uptake value (SUV) > 2.5 was automatically obtained from the 3D volume that included the ankle joint. MTV represented the active extent of trauma-related joint disease, similar to the metabolic tumor volume that is widely used in the oncology field. Within these MTVs, maximum and mean SUV (SUVmax and SUVmean) were measured. The total lesion activity (TLA) was also determined; this concept was adopted from total lesion glycolysis (TLG), and was the product of the SUVmean and MTV. These parameters express additional information about disease activity in terms of disease extent, represented by MTV [17].

### Statistical analysis

Data were analyzed using SPSS 23.0 statistical software (SPSS Inc., Chicago, IL). An independent *t*-test was used to compare hot-uptake parameters of 18F-NaF PET/CT between the fracture group and non-fracture group. The relationship between ankle ROM and hot-uptake parameters of 18F-NaF PET/CT was analyzed using Pearson’s correlation coefficient. *P* < .05 was considered to indicate statistically significant differences.

## Results

**Fig. 1:**
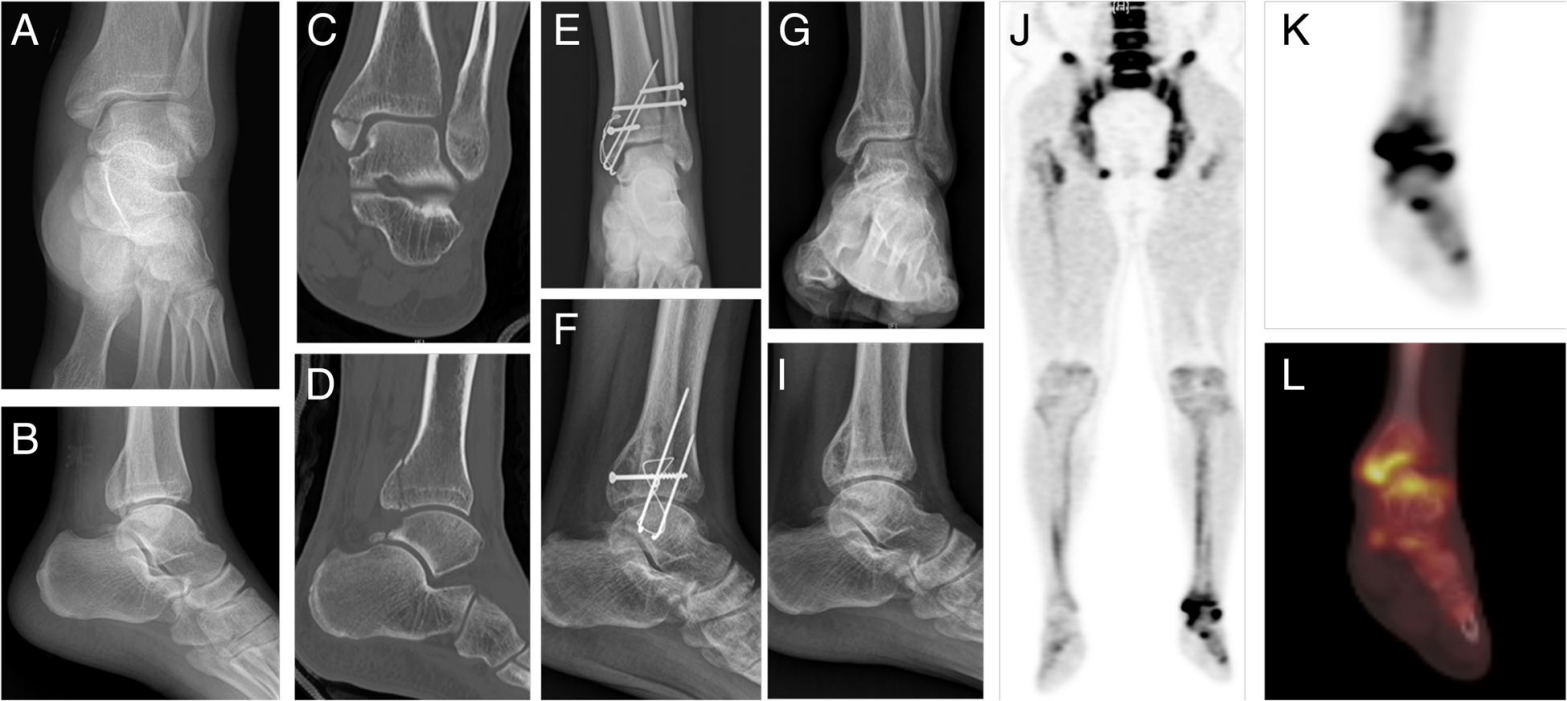
Radiological and ^18^F-Sodium fluoride (18F-NaF) positron emission tomography-computed tomography (PET/CT) images of a 37-year-old male patient who had a fracture of the left medial and posterior malleolus of the tibia. **a**-**d** Initial images of plain radiographs and computed tomography (CT); **a** ankle anterior-posterior view, **b** ankle lateral view, **c** ankle CT coronal view, **d** ankle CT lateral view. Note that the fracture involved the talocrural (tibiotalar) joint. **e**, **f** Plain radiographs after internal fixation with a metal pin. **g**-**i** Plain radiographs performed at 17 months after injury presents pin removal status and osteoporotic changes around the ankle joint, but does not provide any information about the degree of ankylosis in the ankle. **j**-**l**) 18F-NaF PET/CT shows hot uptake around the talocrural joint near the initial fracture area

**Fig. 2:**
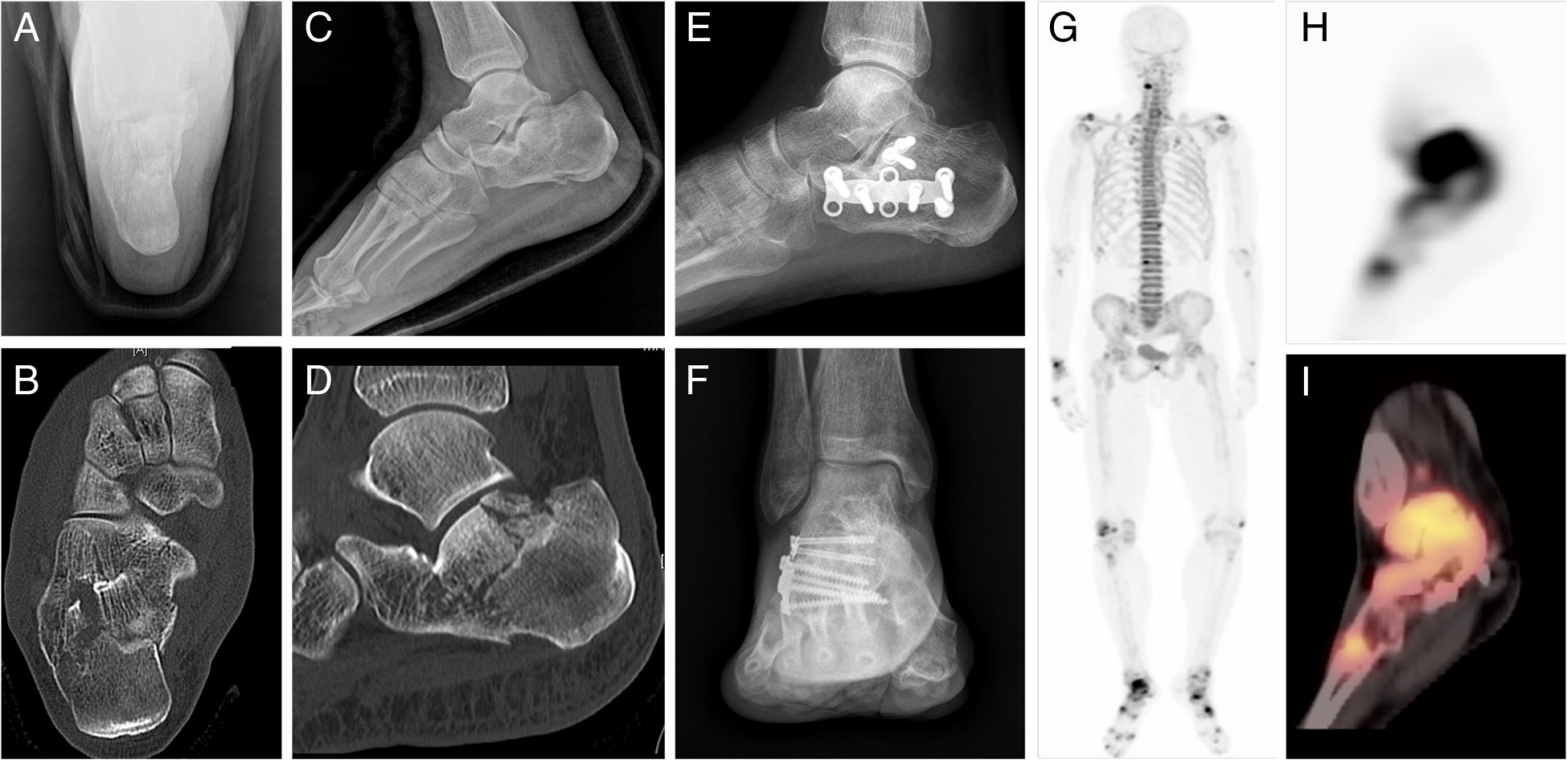
Radiologic and ^18^F-sodium fluoride (18F-NaF) positron emission tomography-computed tomography (PET/CT) images of a 53-year-old male patient who had a right calcaneus fracture and underwent operation with plate-screw fixation. **a**-**d** Initial images of plain radiographs and computed tomography (CT); **a** calcaneal anterior-posterior view, **b** calcaneal CT axial view, **c** ankle lateral view, **d** calcaneal CT lateral view. Note that the fracture did not involve the talocrural (tibiotalar) joint. **e**, **f** Plain radiographs performed at 9 months after injury presents plate-screw fixation, but does not give any information about talocrural joint pathology and the degree of ankylosis in the ankle. **g**-**i** 18F-NaF PET/CT shows hot uptake around the talocrural joint away from the initial fracture and plate-screw fixation area

The median period from injury to performing 18F-NaF PET/CT was 290 days (range: 180–2396). The causes of injury were as follows: fracture (*N* = 95; tibia, and/or fibular, and/or calcaneus), Achilles tendon rupture (*N* = 12), complex regional pain syndrome (*N* = 2), ligament injury (*N* = 12, anterior talofibular ligament, and/or calcaneofibular ligament, and/or deltoid ligament). Quantitative analysis of 18F-NaF PET/CT revealed that 113 of 121 patients had hot uptake (SUVmax > 2.5) in the ROI (region of interest) of the ankle, whereas the remaining 8 did not. All 8 patients who did not show hot uptake were in the non-fracture group. Representative cases are presented in Figs. 1 and 2.

The SUVmax (13.31 ± 9.42 vs. 5.33 ± 3.24, *p* < .01), SUVmean (5.92 ± 4.94 vs. 3.07 ± 1.68, *p* < .05), and TLA (268.03 ± 349.76 vs. 73.05 ± 106.20, *p* < .05, Table 2) values were higher in the fracture than in the non-fracture group. The fracture group also showed a tendency for higher values in MTV than non-fracture group without statistical significance (*p* = .068). A more limited ROM of the ankle was correlated with higher hot-uptake parameters of 18F-NaF PET/CT: SUVmax (*ρ* = − 0.335, *p* < .01), SUVmean (*ρ* = − 0.343, *p* < .01), MTV (*ρ* = − 0.252, < 0.05), and TLA (*ρ* = − 0.305, *p* < .01, Table 3). In a subgroup analysis, the fracture group revealed similar results: SUVmax (*ρ* = − 0.336, *p* < .01), SUVmean (*ρ* = − 0.354, *p* < .01), and TLA (*ρ* = − 0.292, *p* < .05), whereas these tendencies were not observed in the non-fracture group (Table 3).

**Table 2:** Comparison of hot uptake parameters of 18F-NaF PET/CT between the fracture group and the non-fracture group

	Fracture (*N* = 95)	Non-fracture (*N* = 26)	*p*-value
SUVmax	13.31 ± 9.42	5.33 ± 3.24	0.001
SUVmean	5.92 ± 4.94	3.07 ± 1.68	0.017
MTV	52.40 ± 67.09	22.86 ± 29.72	0.068
TLA	268.03 ± 349.76	73.05 ± 106.20	0.019

Values represent the mean ± standard deviation

18F-NaF PET/CT, 18-Fluorine sodium fluoride bone positron emission tomography-computed tomography; *MTV* metabolic target volume, *SUVmax* maximum standardized uptake value, *SUVmean* mean standardized uptake value, *TLA* total lesion activity

**Table 3:** Correlation coefficient between ankle ROM and hot uptake parameters of 18F-NaF PET/CT

	Total (*N* = 121)	Fracture (*N* = 95)	Non-fracture (*N* = 26)
SUVmax	- 0.335**	- 0.336*	- 0.102
SUVmean	- 0.343**	- 0.354**	- 0.059
MTV	- 0.252*	- 0.223	- 0.317
TLA	- 0.305**	- 0.292*	- 0.265

Values represent Pearson’s correlation coefficient. * *P* < 0.05, ** *P* < 0.01

18F-NaF PET/CT, 18-Fluorine sodium fluoride bone positron emission tomography-computed tomography; *MTV* metabolic target volume, *ROM* range of motion, *SUVmax* maximum standardized uptake value, *SUVmean* mean standardized uptake value, *TLA* total lesion activity

## Discussion

According to our retrospective study of 18F-NaF PET/CT findings, most patients with limited ROM after ankle trauma (113/121 = 93.4%) showed hot uptake around the ankle joint. However, several cases (8/26 = 30.8%) in the non-fracture group did not reveal hot uptake on PET/CT, and even if some hot uptake was observed, there was no statistical significant correlation between the degree of hot uptake and the limitation of ankle ROM (Table 3). In contrast, 100% patients in the fracture group (*N* = 95) showed hot uptake around the ankle joint, and the quantitative parameters (SUVmax and SUVmean, but not MTV) were statistically significantly correlated with the limitation of ankle ROM (Table 3). Therefore, it can be assumed that the “intensity” of hot uptake was more relevant than the “spread” of hot uptake. These results suggest that 18F-NaF bone PET/CT can be an objective imaging tool for evaluating ankle joint disability in patients with ankle fracture.

In our study, higher hot uptake parameters of 18F-NaF PET/CT were correlated with more limited ROM of the ankle joint. As 18F-NaF is a bone imaging agent, the higher uptake of this agent in patients with greater immobility and soft tissue rigidity requires some explanation. Tatsuya et al. reported arthroscopic mobilization of the wrist by removing a septum that was assumed to have developed after trauma [8]. They reported that this septum could not be detected by plain radiographs, magnetic resonance image, or CT. Moreover, they reported that arthroscopic surgery could not restore the full ROM, and that immature chondrocytes were observed around the fibers. Furthermore, the joint contracture could be produced by altered length-tension relationships and neuromuscular mechanisms after fractures [18]. During the period of ankle fixation, some changes in neuromuscular conditions occur due to immobilization to protect against overstretching of the fragile musculature around the ankle joint [18]. In light of these findings about soft tissue contracture after joint trauma, the higher bone uptake of 18F-NaF in the patients with greater ROM limitation may be attributable to greater immobility caused by tighter contracture. Greater immobility is expected to cause more disuse osteoporosis. Disuse osteoporosis involves reversible bone loss, associated with increased osteoclastic bone resorption in the mechanically unloaded bone [9]. The bones affected by disuse osteoporosis are associated with eventually greater turnover of bones and show a high radiopharmaceutical uptake reflecting osteoblastic activity [19]. Greater joint contracture is therefore expected to cause more disuse osteoporosis, and consequently more osteoblastic activities which are represented as radiopharmaceutical uptake in 18F-NaF PET/CT [14, 20]. Increased osteoblastic activities may cause hypertrophic sclerosis which leads pseudoarthroses and chronic disability.

Fluorodeoxyglucose (FDG) PET/CT indicates the tissue glucose metabolic rate, and thus, when it is performed for trauma-related arthritis, it mainly reflects the inflammatory process in the soft tissue in the joint [21]. However, FDG uptake is not sensitive for detection of bone formation or periosteal bone reaction. Although conventional bone scintigraphy is known to have high sensitivity for detection of bone reaction, its resolution is not high and exact localization of hot-uptake lesions is not possible [22]. Recently, SPECT/CT using ^99m^Tc-labeled agents is available in some hospitals, but has relatively low image resolution and a long scan-time. Therefore, we evaluated the use of 18F-NaF bone PET/CT to validate bone reaction in the complicated bony structure of the ankle, because of its high resolution and sensitivity, as well as its ability to allow exact lesion localization.

Trauma involving the ankle joint can occur anywhere, such as work-related injury, a road traffic accident, or slipping in a public area. Although patients typically have significant improvement after appropriate initial treatment, they often still have residual physical impairment several years after the injury [23–25]. Because these cases can be related to medicolegal or various compensation issues, objective evaluation of permanent impairment is very important. Although ROM appears to be a suitable method for evaluating impairment of the ankle joint [26, 27], it may be subject to variation, because patients may complain of pain during motion at different times during the examination, and may be deliberately uncooperative and inconsistent. With such inconsistency, ROM assessment cannot be used as a valid parameter in impairment evaluations [26]. Under these conditions, 18F-NaF bone PET/CT findings could be used as useful information for increasing the reliability of impairment evaluations. The results of our study suggest that hot uptake of 18F-NaF bone PET/CT reflects greater impairment, even if the ankle ROM assessment is somewhat inconsistent. However, it should not be concluded that less hot uptake of 18F-NaF during bone PET/CT indicates deception by the patients during impairment evaluation.

Our study had some limitations. Because this study was a pilot study, no control group was employed, which may be considered a limitation. A no-ROM limitation group, even after ankle injury, would facilitate understanding of the role of 18F-NaF bone PET/CT in permanent impairment evaluation. However, it is very difficult to recruit patients who have no physical impairment several months after ankle injury, as they would usually not visit the hospital regularly and there would be no reason to undergo expensive imaging examinations, such as PET/CT. The numbers between the fracture group and non-fracture group were much differed in this study (95 vs. 26). The reason for this is that the study was not a prospective study, and it is the patients in fracture group are more likely to have a disability because the impact of accident may be greater than non-fracture group. Moreover, we did not consider the effect of other factors that may affect hot uptake of 18F-NaF bone PET/CT, such as age, sex, and amount of physical activity of the patients. Further evaluation was not performed for several conditions or factors that could affect bone metabolism, such as diabetes mellitus, osteoporosis, and the use of steroids. Further studies are necessary to accumulate more 18F-NaF bone PET/CT data in the context of ankle injury in order to establish a cut-off value for adjudication of permanent impairment.

## Conclusions

18F-NaF PET/CT can provide considerable information in impairment evaluations of limited ankle ROM, particularly in patients with fracture around the ankle joint. Therefore, it can be assumed that the “intensity” of hot uptake was more relevant than the “spread” of hot uptake.

## Abbreviations

18F-NaF: ^18^F-sodium fluoride
^99m^Tc: Technetium-99 m
CT: Computerized tomography
FDG: Fluorodeoxyglucose
HD: Ultra-high definition
MMI: Maximum medical improvement
MTV: Metabolic target volume (MTV)
NaI (Tl): Thallium-doped sodium iodide
PET: Positron emission tomography
PTA: Posttraumatic osteoarthritis
ROM: Limitation of range of motion
SUV: Standardized uptake value
TLA: Total lesion activity;
TLG: Total lesion glycolysis
TOF: Time of flight (TOF)
